# Recyclable and Scalable Cellulose/SiO_2_ Fiber Enabling Thermal and Moisture Comfort

**DOI:** 10.3390/polym18151888

**Published:** 2026-07-31

**Authors:** Xinxin Li, Chaoqun Ji, Youjia Yang, Kaisheng Zeng, Lihui Chen, Jianguo Li, Yonghao Ni, Bin Chen

**Affiliations:** 1 National Forestry and Grassland Administration Key Laboratory of Plant Fiber Functional Materials, College of Material Engineering, Fujian Agriculture and Forestry University, Fuzhou 350002, Chinajianguolicn@fafu.edu.cn (J.L.); 2 Department of Chemical Engineering, University of New Brunswick, Fredericton, NB E3B 5A3, Canada

**Keywords:** cellulose, thermal and moisture comfort, hierarchical interface-pore structure, recyclable, biodegradable

## Abstract

Developing sustainable and scalable personal thermal management textiles that simultaneously provide radiative cooling, moisture comfort, and responsible end-of-life management remains challenging. Here, we report a sustainable, scalable, and recyclable bamboo dissolving pulp-derived cellulose/SiO_2_ fiber (CSF), fabricated by a wet-spinning process involving the dissolution and regeneration of cellulose and nano-SiO_2_. The resultant CSF exhibits a hierarchical interface-pore structure, which enhances solar scattering (up to 94.56% in 0.4–1.0 μm) by Mie scattering of nano-SiO_2_ particles and multiple scattering at micro- and nanopore-induced air/cellulose/SiO_2_ interfaces. By coupling high mid-infrared emissivity of 94.8% (8–13 μm), the CSF demonstrates average daytime sub-ambient cooling of 9.5 °C under hot and humid summer conditions. More importantly, the CSF presents a multiscale water-transport network that integrates molecular water capture (–OH groups), capillary infiltration (nanoscale interfaces between nano-SiO_2_ and cellulose), and liquid spreading and evaporation (interconnected microchannels between fibers), which realizes larger liquid diffusion area and water-vapor transmission rate (7.55 cm^2^ and 175.48 g m^−2^ 24 h^−1^), compared to commercial cotton and polyester. In addition, the CSF demonstrates desirable soil-biodegradation capability, while the feasibility of closed-loop reuse is demonstrated through a single recycling cycle, supporting environmentally friendly wearable cooling textiles. The wet-spinning strategy paves the way for the construction of sustainable, scalable and recyclable fiber for thermal- and moisture-comfort textiles.

## 1. Introduction

With the acceleration of global industrialization and intensified anthropogenic activities, large quantities of greenhouse gases have been continuously released into the atmosphere in recent years, thereby exacerbating global warming and significantly increasing the risk of heat-related health problems [1,2,3]. To alleviate thermal discomfort under hot conditions, personal thermal management (PTM) has been proposed as an effective strategy for dynamically regulating human thermal balance [4,5,6,7,8]. Among the various PTM approaches, passive daytime radiative cooling (PDRC) is widely regarded as an effective route toward passive heat dissipation, because it enables net heat rejection by reflecting solar radiation in the 0.3–2.5 μm range while simultaneously emitting thermal radiation through the 8–13 μm atmospheric window [9]. Accordingly, efficient PDRC materials generally require both high solar reflectance and strong mid-infrared emissivity [10,11,12,13]. Against this background, the concept of wearable radiative-cooling textiles can be traced back to the radiative human body cooling strategy proposed by Hsu et al. [14], while the hierarchical-morphology metafabric later reported by Zeng et al. marked a key milestone in extending this concept to scalable passive daytime radiative-cooling fabrics [15]. Along this line, a variety of polymeric materials have been developed for wearable PDRC applications [16,17,18,19,20], including polyethylene, polyurethane, polyvinylidene fluoride, and polytetrafluoroethylene [21]. These materials can effectively reflect solar radiation while allowing body heat to dissipate to the surroundings in the form of mid-infrared radiation. Despite substantial progress in cooling performance through enhanced solar reflectance and mid-infrared emissivity, most reported materials remain petroleum-derived and nondegradable, raising persistent concerns about environmental sustainability, biosafety, and end-of-life disposal [22,23,24].

Cellulose has emerged as an ideal material platform for sustainable radiative-cooling textiles owing to its renewability [25,26,27,28,29], biodegradability, hydrophilicity, and intrinsic mid-infrared emissivity [30,31,32]. These characteristics make cellulose particularly attractive for wearable thermal management [33,34]. Representative studies have already demonstrated the promise of cellulose-based textiles in personal thermal management. For example, Li et al. developed a biomimetic cellulose fabric for sustainably and durably cooling the human body [35], while Zhong et al. reported a hierarchically structured cellulose-based fabric for outdoor personal thermal management [36]. These cellulose-based textile studies demonstrate that cellulose materials can be engineered into radiative-cooling textiles with effective solar reflection, mid-infrared heat dissipation, and practical personal thermal management capability. Nevertheless, rapid moisture dissipation in cellulose-based radiative-cooling textiles remains challenging under realistic wearing conditions, especially in hot and humid regions worldwide, where high temperature and high relative humidity impose simultaneous demands on thermal regulation and moisture management [37,38,39].

Here, we reported a sustainable, scalable and recyclable cellulose/SiO_2_ fiber (CSF) fabricated by a green wet-spinning process [40]. Similar to the preparation of conventional lyocell and viscose fibers, natural cellulose is first dissolved in an ionic-liquid solvent [41], followed by the incorporation of nano-SiO_2_ particles to form a cellulose/SiO_2_ spinning solution. Water is then introduced as the antisolvent to regenerate the cellulose/SiO_2_ matrix. This wet-spinning strategy enables the fabrication of CSF while also demonstrating desirable scalability and sustainability, thus providing a viable route toward wearable textiles. Benefiting from this dissolution–regeneration process, nano-SiO_2_ can be uniformly dispersed within the cellulose matrix, thereby generating a hierarchical interface-pore structure and exposing rich chemical groups (such as C-O-C and Si-O), thus imparting a high reflectance of 94.56% in the 0.4–1.0 μm region and a mid-infrared emissivity of 94.8% in the 8–13 μm atmospheric window. Consequently, the CSF exhibits excellent radiative-cooling performance, achieving an average daytime sub-ambient cooling of 9.47 °C below ambient temperature. Under realistic wearable conditions, the CSF-covered skin region is 5.4 °C cooler than bare skin and 4.2 °C cooler than the commercial cotton. The abundant –OH groups of cellulose and the Si–OH groups of SiO_2_ promote water uptake via hydrophilic interactions [42], while the hierarchical interface-pore structure of the CSF provides interconnected micro- and nanochannels for capillary-driven moisture transport and vapor release. As a result, the CSF exhibits efficient water/moisture-management capability, with a high water-vapor transmission rate (WVTR) of 175.48 g m^−2^ 24 h^−1^. After use, the cellulose framework of the CSF undergoes soil biodegradation, or used CSF can also be dissolved in ionic liquid under heating to form a cellulose/SiO_2_ composite solution for closed-loop reuse. By integrating radiative cooling, evaporative moisture management, washing durability, soil biodegradation, and dissolution–regeneration capability, followed by closed-loop reuse, this sustainable, scalable, and recyclable CSF provides a lifecycle-responsible platform for next-generation wearable cooling textiles, particularly in hot and humid regions worldwide.

## 2. Materials

Commercial bamboo dissolving pulp (degree of polymerization of 511, α-cellulose content of 91%, ash content ≤0.08%, lignin content <1%, and ISO brightness of 89.6%) as the natural cellulose source is obtained from an enterprise in Fuzhou, China. Spherical silica nanoparticles (nano-SiO2, average diameter: 500 nm) are purchased from Aladdin Biochemical Technology Co., Ltd (Shanghai, China). The ionic liquid of 1-allyl-3-methylimidazolium chloride ([AMIM]Cl) is obtained from Lanzhou Yulu Fine Chemical Co., Ltd. (Lanzhou, China).

### 2.1. Preparation of CSF

CSF is fabricated via a continuous wet-spinning process. The mass ratio of cellulose:nano-SiO_2_:[AMIM]Cl in the spinning dope is 1:1:10. Nano-SiO_2_ and [AMIM]Cl are first mixed at 110 °C, followed by the addition of dissolving pulp and further stirring for 50 min to obtain a homogeneous spinning dope. Subsequently, the dope is extruded through a micro-syringe pump into a water coagulation bath. The nascent fibers undergo pre-stretching, pass through a secondary water bath, and are then collected. To thoroughly extract the residual ionic liquid, the collected fibers are immersed in fresh water to replace the solvent of ionic liquid. After thorough washing to remove the residual ionic liquid, the regenerated CSF is dried in a temperature-controlled chamber at 25 °C for 6 h until a stable mass is reached (residual moisture content: 4.75 wt%, wet basis). The continuous CSF is woven on a loom and used as both warp and weft yarns. The resulting fabric has an orthogonal warp–weft interlacing structure, a warp density of 10 ends cm^−1^, a weft density of 30 picks cm^−1^ and a thickness of 4.0 mm.

### 2.2. Characterization

The morphology of the samples is characterized using a field-emission scanning electron microscope (FE-SEM; SU8010, Hitachi High-Technologies Corporation, Tokyo, Japan). Prior to observation, all samples are sputter-coated with gold for 180 s. Energy-dispersive spectroscopy (EDS) analysis is conducted on a Sigma 300 microscope (Carl Zeiss Microscopy GmbH, Jena, Germany) equipped with an XFlash 6|60 detector (Bruker Nano GmbH, Berlin, Germany). The hydrodynamic particle-size distribution of the SiO_2_ is characterized by dynamic light scattering using a Zetasizer Nano ZS90 particle-size analyzer (Malvern Instruments Ltd., Malvern, UK). Data is recorded using an OM-CP-OCTPRO data logger (OMEGA Engineering, Inc., Norwalk, CT, USA). The ambient temperature is monitored with a thermocouple placed inside the testing enclosure and shielded with a polyethylene (PE) film, so as to reflect the local thermal environment relevant to the outdoor evaluation of CSF. The solar reflectance of the samples is measured using a Lambda 750 UV–Vis–NIR spectrophotometer (PerkinElmer, Inc., Shelton, CT, USA) equipped with an integrating sphere. For the laboratory washing-stability test, each cycle consists of washing the CSF fabric for 15 min, and its solar reflectance is measured after the designated number of cycles. The test is conducted under laboratory washing conditions to evaluate the reflectance retention of CSF after repeated laboratory machine washing. The reflectance r(λ) and transmittance t(λ) in the mid-infrared (MIR) region are measured using a Nicolet iS50 FTIR spectrometer (Thermo Fisher Scientific Inc., Waltham, MA, USA). The water contact angle is measured at room temperature using a DSA30 analyzer (KRÜSS GmbH, Hamburg, Germany). The liquid-spreading and vertical-wicking tests are conducted according to AATCC TM198 [43] and AATCC TM213 [44], respectively. The water-vapor transmission performance of the textile samples is evaluated in accordance with ISO 15496:2018 [45], and the results are expressed as the water-vapor transmission rate (WVTR). The crystalline structure of the samples is measured based on the X-ray diffraction system (XRD, MiniFlex II, Rigaku Corporation, Tokyo, Japan) at room temperature. For the soil-burial test, the samples are buried outdoors in natural soil at a depth of approximately 20 cm for 150 days, and their macroscopic changes are recorded. For the reprocessing experiment, the recovered CSF is mixed with [AMIM]Cl at a mass ratio of 1:5 and stirred at 110 °C for 30 min to obtain a homogeneous spinning dope, and is subsequently re-spun to harvest the recycled CSF following the original wet-spinning procedure.

## 3. Results and Discussion

**Figure 1:**
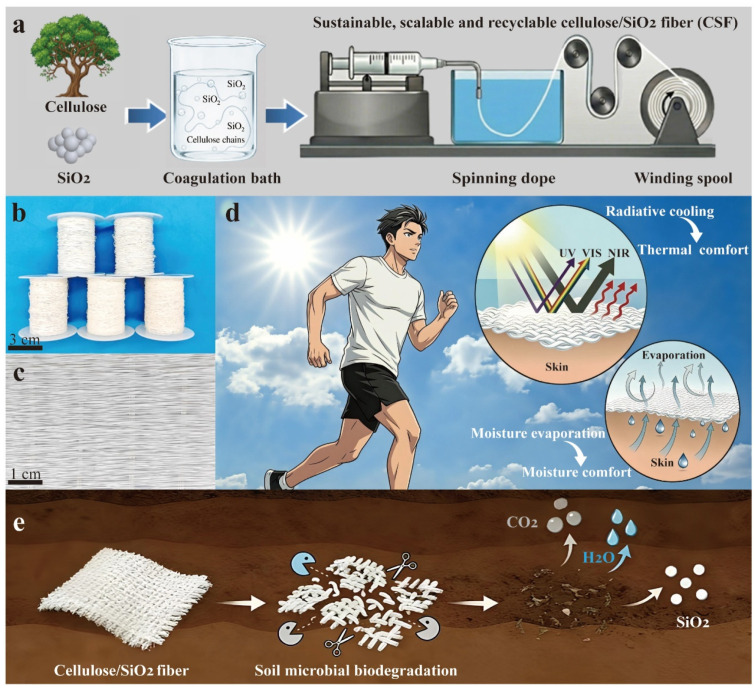
Preparation of sustainable and scalable cellulose/SiO_2_ fiber. (**a**) A schematic illustration of the green wet-spinning process for preparing CSF, including the cellulose/SiO_2_ precursor system, spinning-dope preparation, fiber regeneration in the coagulation bath, and continuous winding. (**b**) A photograph of the continuously collected CSF. (**c**) A photograph of the CSF woven into the textile. (**d**) A schematic illustration of the mechanism of thermal and moisture comfort of CSF, showing radiative cooling through solar reflection and mid-infrared heat emission, together with moisture evaporation enabled by sweat evaporation at the skin–CSF interface. (**e**) An illustration of the end-of-life biodegradation pathway of the CSF in soil, where the cellulose component undergoes soil microbial biodegradation accompanied by the release of CO_2_ and H_2_O, while nano-SiO_2_ is returned to the natural environment as an environmentally benign inorganic component.

Continuous CSF is fabricated by a green wet-spinning process (Figure 1a). The resulting continuous fibers are subsequently woven into a thermal- and moisture-comfortable textile (Figure 1b,c). Moreover, the fiber diameter can be tuned by selecting spinning needles with different inner diameters, such as 100, 200, and 300 μm (Figure S1) [46,47,48], indicating good process controllability of the wet-spinning route. The resulting CSF also exhibits sufficient mechanical robustness for textile fabrication and practical use. A single fiber can be tied into a knot without fracture (Figure S2), suspend a 500 g weight (Figure S3a), and achieve an ultimate tensile stress exceeding 60 MPa (Figure S3b), confirming adequate flexibility and strength for continuous collection, weaving, and handling. These results indicate that the present fabrication strategy not only enables the preparation of CSF, but also provides a viable route toward mechanically reliable wearable textiles. As further summarized in Figure S3c,d, CSFs with diameters of 0.14, 0.23, 0.32, and 0.48 mm exhibited maximum tensile stresses of 62.71, 57.65, 53.32, and 39.79 MPa, respectively. Decreasing the fiber diameter increases the tensile strength and stiffness but reduced the elongation at break.

**Figure 2:**
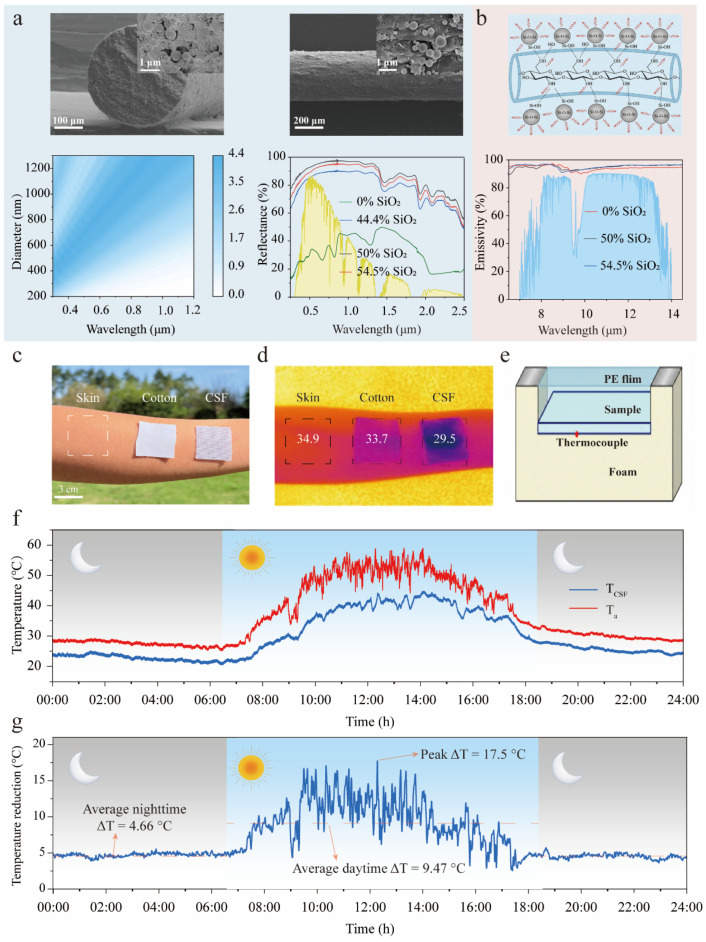
The structural, light regulation, and thermal-comfort performance of CSF. (**a**) Cross-sectional and surface morphologies of the CSF, including simulated scattering intensity as a function of particle diameter and wavelength, and the solar reflectance spectra of samples with different nano-SiO_2_ contents (0%, 44.4%, 50%, and 54.5%). (**b**) The mid-infrared emissivity spectra of the samples. (**c**) A photograph of the outdoor skin-attached cooling test. (**d**) An infrared thermal image comparing skin, cotton, and the CSF. (**e**) A schematic illustration of the outdoor temperature measurement setup. (**f**) Continuous 24 h temperature profiles of the CSF (T_CSF_) and ambient temperature (T_a_) under outdoor conditions (Fuzhou, China, 26 August 2025). (**g**) Temperature reduction in the CSF over 24 h, showing an average daytime Δ*T* of 9.47 °C, an average nighttime Δ*T* of 4.66 °C, and a peak Δ*T* of 17.5 °C.

The structural characteristics of the CSF provide the basis for its light regulation. As shown in Figure 2a, the CSF exhibits a hierarchical interface-pore morphology in which nano-SiO_2_ particles are incorporated into the cellulose framework. The introduced micro-/nanoscale interface-pore structure creates numerous air/cellulose/SiO_2_ interfaces with refractive-index contrast, thereby enhancing multiple scattering of incident solar radiation and improving the reflectance of the CSF. The EDS result confirms the homogeneous distribution of C, O, and Si across the CSF cross-section, demonstrating the uniform spatial distribution of the nano-SiO_2_ in the CSF system (Figure S4). The XRD patterns further reveal that CSF retains the characteristic diffraction features of regenerated cellulose while exhibiting SiO_2_-related diffraction signals, which are consistent with the SiO_2_ reference pattern (JCPDS/PDF No. 99-0088), further supporting the successful incorporation of SiO_2_ into the cellulose fiber matrix (Figure S5). XPS analysis further confirms the incorporation of nano-SiO_2_ into the CSF. Compared with pure cellulose fiber (CF), CSF exhibits additional Si 2p and Si 2s signals in the survey spectrum. In the high-resolution O 1s spectrum [48,49], CF shows a dominant C–O-related oxygen component at 532.2 eV, while CSF can be deconvoluted into Si–O-related oxygen at 532.4 eV, C–O-related oxygen at 532.9 eV [49], and an interfacial C–O–Si-related oxygen component at 531.7 eV (Figure S6) [50]. Moreover, FDTD simulations show that nanoscale SiO_2_ particles generate strong scattering in the solar spectral region, supporting the particle-size design for optical-reflectance enhancement (Figure S7). The strong scattering predicted near a particle diameter of 500 nm guided the selection of commercial SiO_2_ with a nominal average diameter of 500 nm for CSF preparation. This strong scattering originates from Mie scattering, because the diameter of nano-SiO_2_ particles is comparable to the wavelength of incident solar radiation, enabling efficient light–particle interactions. DLS analysis showed that the commercial SiO_2_ had an intensity-weighted distribution of approximately 342–712 nm, and focuses on the diameter of 500 nm (Figure S8). Accordingly, the FDTD model represents a size-selection approach, while the actual particle-size distribution may broaden Mie scattering and affect particle packing, interfacial structure, pore formation, and the resulting properties of CSF. Consistent with these structural and simulation results, the incorporation of nano-SiO_2_ markedly enhances the reflectance of the CSF. At a nano-SiO_2_ content of 50 wt% based on the combined dry mass of cellulose and nano-SiO_2_, corresponding to a cellulose: nano-SiO_2_ mass ratio of 1:1, the CSF achieved an average reflectance of 94.56% in the 0.4–1.0 μm region (Figure 2a). Meanwhile, the intrinsic C-O vibrational absorption of cellulose and the Si–O–Si vibration of nano-SiO_2_ jointly contribute to the high mid-infrared emissivity of the CSF, reaching 94.77% (Figure 2b). These results indicate that the cellulose framework and uniformly incorporated nano-SiO_2_ particles synergistically enable strong scattering across the solar spectrum and efficient mid-infrared thermal emission [51]. The surface hydroxyl and silanol groups primarily govern initial wetting, whereas intrafiber pores and interfiber channels facilitate capillary infiltration and long-range liquid transport, respectively; these roles are consistent with the contact-angle, spreading, wicking, and WVTR results.

The favorable optical properties of CSF further translate into practical outdoor cooling performance. The wearable cooling effect is first evaluated using an outdoor skin-attached test under direct sunlight (Figure 2c). Infrared thermal imaging shows that the CSF-covered skin region exhibits the lowest temperature of 29.5 °C, compared with 34.9 °C for bare skin and 33.7 °C for the cotton-covered region (Figure 2d). This corresponds to a temperature reduction of 5.4 °C to bare skin and 4.2 °C to cotton, confirming the effective wearable cooling capability under realistic outdoor conditions.

To further quantify the cooling performance, continuous temperature monitoring is performed using the setup shown in Figure 2e. CSF is placed on a thermally insulated platform under natural sunlight to minimize conductive heat input from the substrate, while temperature probes are used to measure the temperature of CSF $\left(T_{CSF}\right)$ and the ambient temperature $\left(T_{a}\right)$. Under the hot and humid summer conditions in southeastern China, CSF maintains a stable sub-ambient temperature over a 24 h period (Figure 2f). The corresponding temperature-reduction curve shows an average daytime Δ*T* of 9.47 °C, an average nighttime Δ*T* of 4.66 °C, and a peak Δ*T* of 17.5 °C (Figure 2g). Supplementary continuous outdoor tests conducted on 15 October and 15 November further confirm the stable daytime cooling performance of CSF, with average daytime Δ*T* values of 8.29 °C and 7.92 °C, respectively (Figure S9). These results demonstrate that the cellulose/SiO_2_ architecture effectively converts its light-regulation capability into stable outdoor radiative-cooling performance.

**Figure 3:**
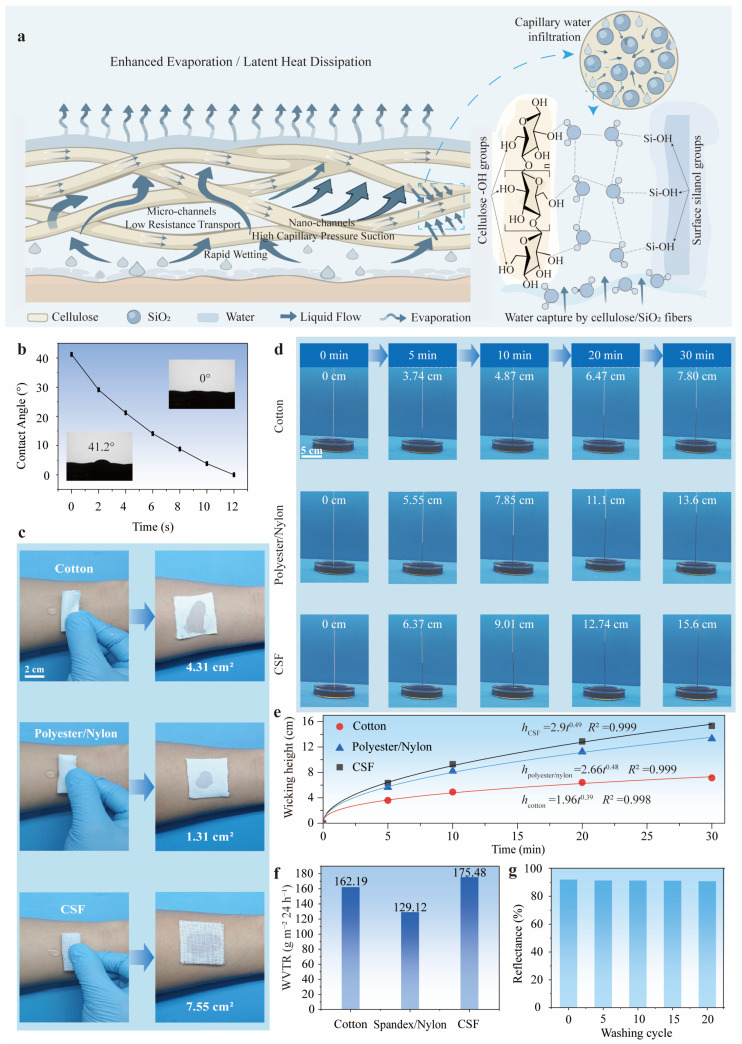
The moisture-management behavior, moisture-comfort performance, and service stability of the CSF. (**a**) A multiscale schematic illustration of water capture, capillary infiltration, liquid transport, and enhanced evaporation in the CSF. (**b**) The dynamic water contact angle of the CSF as a function of time. (**c**) Photographs showing the liquid diffusion behavior of cotton, polyester/nylon, and CSF under simulated perspiration conditions, together with the corresponding diffusion areas. (**d**) Time-dependent photographs of vertical-wicking behavior for cotton, polyester/nylon, and CSF over 30 min. (**e**) Wicking height as a function of time for different fibers, together with power-law fitting curves. (**f**) The water-vapor transmission rates (WVTRs) of cotton, polyester/nylon, and CSF. (**g**) The solar reflectance of the CSF after repeated washing cycles.

Efficient moisture management is essential for maintaining moisture comfort in wearable cooling materials. As illustrated in Figure 3a, CSF forms a multiscale water-transport network that couples molecular water capture, capillary infiltration, liquid spreading, and evaporation. At the molecular level, the –OH groups on cellulose and the Si–OH groups on the nano-SiO_2_ promote water uptake through hydrophilic interactions [52]. Within the fibers with a hierarchical interface-pore structure, nanochannels and nanoscale interfaces between nano-SiO_2_ and the cellulose matrix generate capillary suction, while interconnected microchannels between CSFs reduce liquid-flow resistance and facilitate rapid liquid transport. This multiscale structure provides a pathway for rapid wetting, capillary-driven water transport, and enhanced evaporation [53].

As shown in Figure 3b, the dynamic contact-angle results show that the water contact angle decreases from 41.2° to 0° within 12 s, indicating rapid water absorption by the interface-pore CSF network. In the simulated perspiration test (Figure 3c), the effective liquid diffusion area of CSF reaches 7.55 cm^2^, which is 75% larger than that of commercial cotton (4.31 cm^2^) and substantially higher than that of commercial polyester/nylon (1.31 cm^2^). Vertical-wicking tests are further conducted to evaluate the capillary-driven liquid transport capability of different textiles. As shown in Figure 3d, the liquid gradually climbs along the fibers from 0 to 30 min, driven by capillary forces within the fiber and interfiber channels. The CSF shows the fastest liquid-rise process and the highest wicking height among all tested fibers. Quantitatively, the wicking height of the CSF reached 6.37 cm at 5 min, 9.01 cm at 10 min, 12.74 cm at 20 min, and 15.6 cm at 30 min, which was substantially higher than that of commercial cotton (7.80 cm) and commercial polyester/nylon (13.6 cm) at 30 min. The wicking kinetics were further fitted using a power-law model, yielding $h_{cotton}=1.96t^{0.39}$, $h_{polyester/nylon}=2.66t^{0.48}$, and $h_{CSF}=2.90t^{0.49}\;$ (Figure 3e). The larger coefficient and exponent of the CSF indicate more efficient capillary-driven moisture transport. In addition, CSF exhibits the highest water-vapor transmission rate, reaching 175.48 g m^−2^ 24 h^−1^, compared with 162.19 g m^−2^ 24 h^−1^ for commercial cotton and 129.12 g m^−2^ 24 h^−1^ for commercial polyester/nylon (Figure 3f). Together, these results demonstrate that the interface-pore architecture of CSF accelerates liquid spreading and capillary transport while facilitating vapor release, thereby improving moisture comfort. Service stability is also critical for wearable cooling materials. As shown in Figure 3g, CSF retains a solar reflectance above 90% after 20 washing cycles, demonstrating good washing durability.

**Figure 4:**
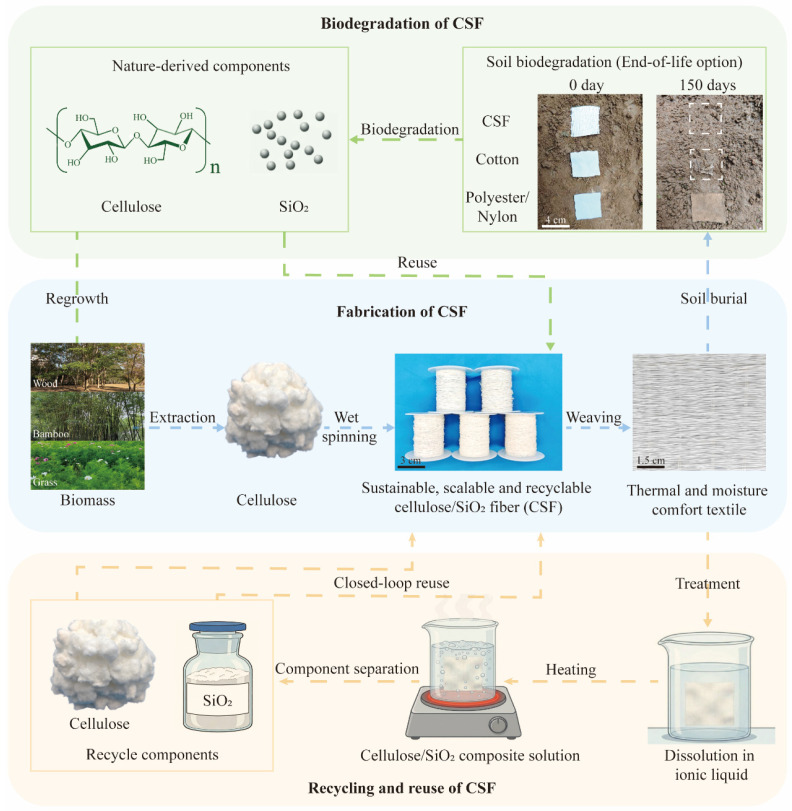
The closed-loop lifecycle of CSF. The schematic summarizes the fabrication process, soil-biodegradation behavior, and post-use recycling pathway of CSF. CSF is composed of cellulose and SiO_2_, in which cellulose is obtained from biomass through extraction. The extracted cellulose with SiO_2_ is processed by wet spinning to produce sustainable and scalable CSF, which is subsequently woven into a thermal- and moisture-comfort textile. As an end-of-life option, soil-burial tests compare the biodegradation behavior of CSF, cotton, and polyester/nylon. In parallel, used CSF can be disintegrated in ionic liquid under heating to form cellulose/SiO_2_ composite solution, followed by component separation to recover cellulose and SiO_2_ for closed-loop reuse (thus manufacturing the secondary CSF).

As shown in Figure 4, CSF is composed of cellulose and nano-SiO_2_, where cellulose is derived from biomass (such as wood, bamboo, and grass) and serves as the renewable organic component, while nano-SiO_2_ acts as the inorganic functional phase. This combination provides the material basis for integrating sustainable sources with radiative-cooling performance.

After utilization, the CSF is subjected to soil burial, and biodegradation occurs through microbial decomposition of the cellulose framework. The hydrophilic cellulose component absorbs moisture and swells, allowing soil microorganisms and cellulolytic enzymes to access the cellulose chains. Enzymatic hydrolysis converts cellulose into soluble sugars, which can be further metabolized by microorganisms into CO_2_, H_2_O, and microbial biomass under aerobic soil conditions. Consequently, the cellulose component gradually decomposes, leading to the macroscopic disintegration of CSF after 150 days. In this process, nano-SiO_2_ is returned to the natural environment as an environmentally benign inorganic component. The soil-burial experiment further evaluates the end-of-life degradation behavior of CSF compared with commercial cotton and commercial polyester/nylon. CSF exhibits complete macroscopic disintegration after 150 days of soil burial, indicating its potential soil biodegradability. In contrast, the commercial 100% cotton plain-woven fabric, with a warp density of 70 ends inch^−1^, a weft density of 60 picks inch^−1^, and a thickness of 0.25 mm, retains visible fragments after 150 days. This difference may be associated with variations in crystallinity, textile structure, thickness, and possible finishing treatments. More importantly, the commercial polyester/nylon shows no obvious morphological changes, indicating its non-degradation.

In addition, the CSF demonstrates desirable post-use recycling and reuse capability. The end-of-life CSF can be deconstructed in ionic liquid under heating treatment, including the re-dissolution of the cellulose phase and re-dispersion of nano-SiO_2_ to form a cellulose/SiO_2_ composite solution. The resulting cellulose/SiO_2_ solution is then available for closed-loop reuse in remanufacturing the secondary CSF. To further verify the functional retention after closed-loop reuse, the optical reflectance and cooling performance of pristine CSF (PCSF) and recycled CSF (RCSF) are compared. As shown in Figure S10a, RCSF retains a high average reflectance of 94.45% in the 0.4–1.0 μm region, which is nearly identical to that of the PCSF (94.56%), corresponding to a reflectance retention of 99.88%. This result indicates that the ionic-liquid-assisted dissolution and re-spinning process does not compromise the solar-reflectance capability of CSF. The outdoor cooling test further confirms the retained radiative-cooling performance after recycling. As shown in Figure S10b, the RCSF maintains an average daytime cooling of 8.02 °C, close to that of PCSF (8.23 °C), demonstrating that CSF preserves an outdoor cooling capability after closed-loop reuse. In conclusion, the design of CSF extends beyond fabrication and wearable use to include both soil biodegradation and component recovery, demonstrating a closed-loop strategy for sustainable wearable thermal and moisture management.

## 4. Conclusions

In summary, a sustainable cellulose/SiO_2_ fiber derived from bamboo dissolving pulp was fabricated by wet spinning to integrate thermal and moisture comfort. During regeneration, solvent–nonsolvent exchange creates hierarchically skinless fibers with a hierarchical interface-pore structure with uniformly distributed nano-SiO_2_, thereby enabling solar scattering, mid-infrared emission, and capillary moisture transport within a single architecture. CSF achieves an average reflectance of 94.56% in the 0.4–1.0 μm region and an average mid-infrared emissivity of 94.8%, delivering stable sub-ambient cooling under outdoor conditions with an average daytime Δ*T* of 9.47 °C. Its multiscale interface-pore network promotes wetting, liquid spreading, wicking, and vapor release, giving a moisture diffusion area 75% larger than that of cotton, a wicking height of 15.6 cm within 30 min, and a WVTR of 175.48 g m^−2^ 24 h^−1^. CSF shows washing durability and soil biodegradation, while the feasibility of closed-loop component recovery is demonstrated through a single recycling cycle, supporting sustainable wearable thermal management. This design offers a fiber-material platform with desirable thermal and moisture comfort, scalability, durability, and lifecycle responsibility [54].

## Data Availability

The original contributions presented in this study are included in the article/Supplementary Material. Further inquiries can be directed to the corresponding authors.

## Supplementary Materials

The following supporting information can be downloaded at https://www.mdpi.com/article/10.3390/polym18151888/s1, Figure S1: Cross-sectional SEM images of CSF with different diameters. The spinning parameters are adjusted to obtain fibers with average diameters of approximately (a) 100 μm, (b) 200 μm, and (c) 300 μm.; Figure S2: Optical photograph demonstrating the flexibility of a CSF, which can be tied into multiple tight knots without fracture.; Figure S3: Mechanical properties of the CSF. (a) Photograph of a single CSF fiber suspending a 500 g standard weight without fracture. (b) Representative stress–strain curves of CSF fibers with different diameters (0.14, 0.23, 0.32, and 0.48 mm). (c) Young’s modulus and (d) elongation at break of CSF fibers with different diameters.; Figure S4: EDS spectrum of CSF, demonstrating the homogeneous spatial distribution of the SiO_2_ in the CSF.; Figure S5: XRD characterization of CF, CSF, and SiO_2_. XRD patterns of cellulose fiber (CF), cellulose/SiO_2_ fiber (CSF), SiO_2_ particles, and the standard SiO_2_ reference pattern JCPDS/PDF #99-0088.; Figure S6: XPS characterization of cellulose fiber (CF) and CSF. (a) Survey XPS spectra of CF and CSF. Both samples show characteristic C 1s and O 1s signals, while CSF additionally exhibits Si 2p and Si 2s signals in the enlarged inset, confirming the successful incorporation of SiO_2_ into the fiber system. (b) High-resolution O 1s spectrum of CF, showing a dominant C–O-related oxygen component centered at 532.2 eV. (c) High-resolution O 1s spectrum of CSF, which can be deconvoluted into Si–O-related oxygen at 532.4 eV, C–O-related oxygen at 532.9 eV, and an interfacial C–O–Si-related oxygen component at 531.7 eV.; Figure S7: FDTD simulated electric field distributions (|E|/|E_0_|) of nano-SiO_2_ (200–650 nm). The optimal Mie scattering resonance is observed at a diameter of 500 nm, justifying its use to maximize solar reflectance.; Figure S8: Intensity-weighted hydrodynamic particle-size distribution of the commercial SiO_2_; dispersion measured by dynamic light scattering.; Figure S9: Supplementary continuous outdoor cooling performance of CSF. (a) Photograph of the outdoor experimental setup used for evaluating the cooling performance. The sample is placed on a thermally insulated platform under natural sunlight. (b, c) Daytime temperature profiles of CSF in Fuzhou, China, on (b) October 15th, 2025 and (c) November 15th, 2025.; Figure S10: Light and outdoor cooling performance retention of recycled CSF. (a) Reflectance spectra of pristine CSF (PCSF) and recycled CSF (RCSF). PCSF exhibits an average reflectance of 94.56% in the 0.4–1.0 μm region, while RCSF retains an average reflectance of 94.45% in the same region, corresponding to 99.88% retention relative to PCSF. (b) Outdoor temperature profiles of ambient air (T_a_), PCSF (T_PCSF_), and RCSF (T_RCSF_) in Fuzhou, China, on October 15th, 2025, from 10:00 to 16:00. RCSF maintains an average daytime cooling of 8.02 °C, close to that of PCSF (8.23 °C), indicating that CSF largely preserves its light-reflectance and radiative-cooling capabilities after closed-loop reuse.
