# Supplementary material for: Recyclable and Scalable Cellulose/SiO2 Fiber Enabling Thermal and Moisture Comfort
Source: Polymers (Basel). 2026 Jul 31;18(15):1888. doi: 10.3390/polym18151888 (PMC13467635; doi:10.3390/polym18151888)
Supplement: Supplementary file 1 [file polymers-18-01888-s001.zip › polymers-4438202-supplementary.pdf]

Supporting Information

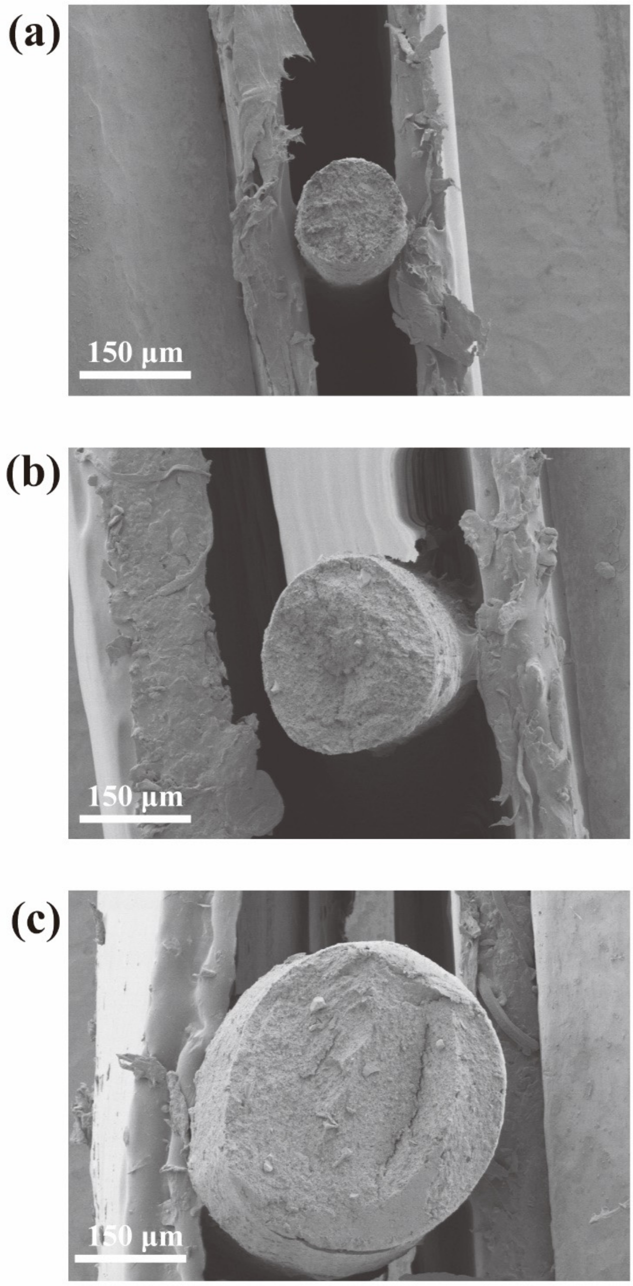

Figure S1. Cross-sectional SEM images of CSF with different diameters. The spinning parameters are adjusted to obtain fibers with average diameters of approximately (a) 100 μm, (b) 200 μm, and (c) 300 μm.

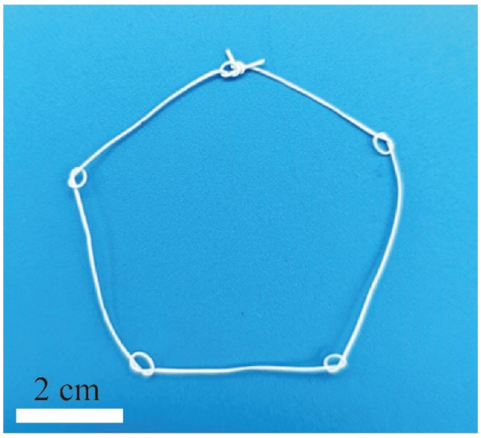

Figure S2. Optical photograph demonstrating the flexibility of a CSF, which can be tied into multiple tight knots without fracture.

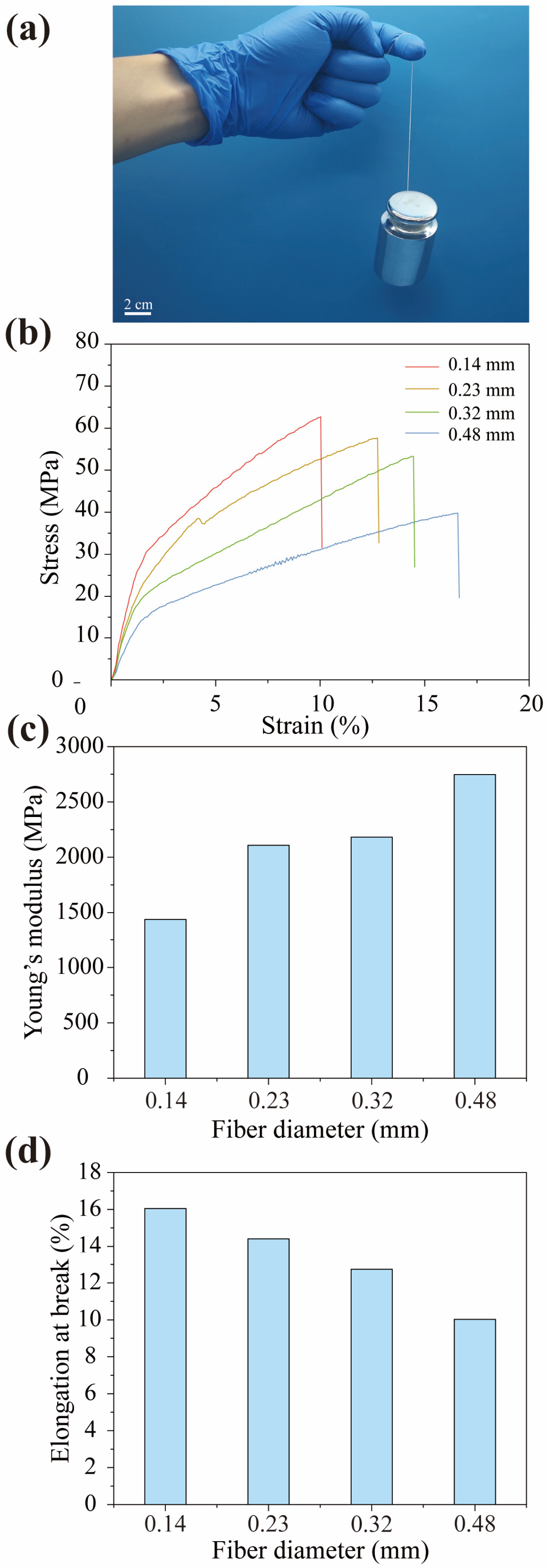

Figure S3. Mechanical properties of the CSF. (a) Photograph of a single CSF fiber suspending a 500 g standard weight without fracture. (b) Representative stress–strain curves of CSF fibers with different diameters (0.14, 0.23, 0.32, and 0.48 mm). (c) Young's modulus and (d) elongation at break of CSF fibers with different diameters.

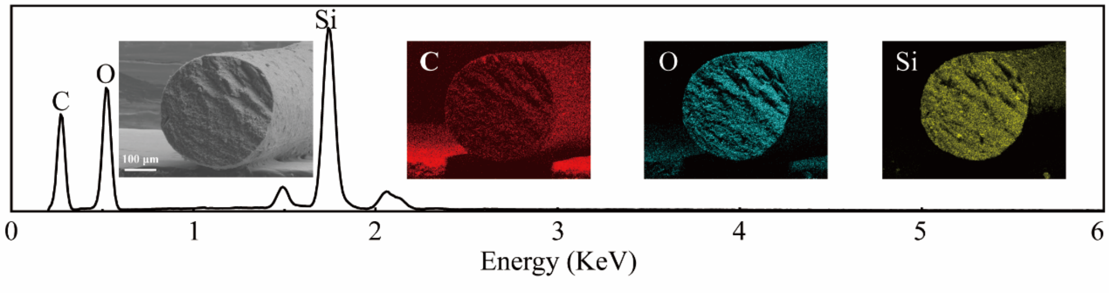

Figure S4. EDS spectrum of CSF, demonstrating the homogeneous spatial distribution of the SiO_2 in the CSF.

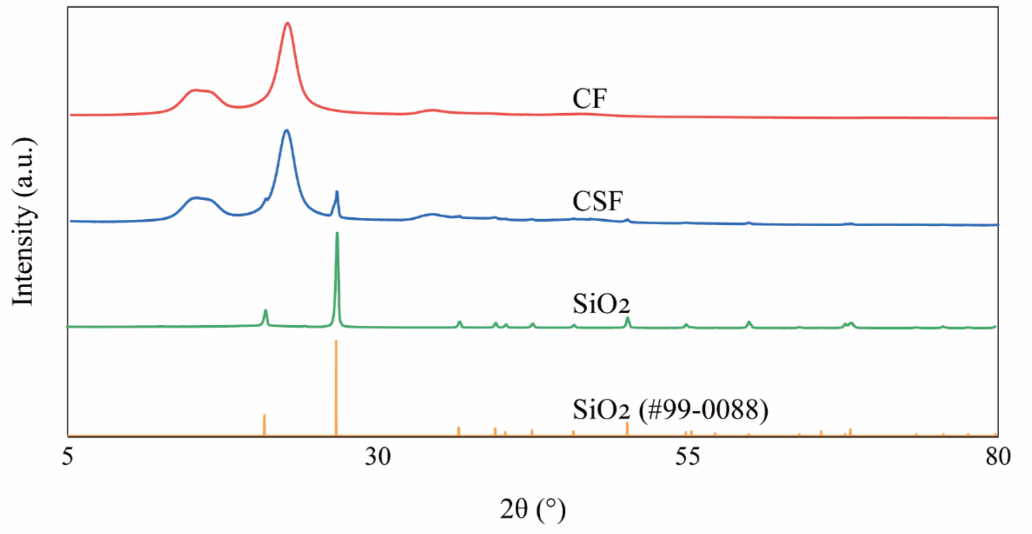

Figure S5. XRD characterization of CF, CSF, and SiO_2. XRD patterns of cellulose fiber (CF), cellulose/SiO_2 fiber (CSF), SiO_2 particles, and the standard SiO_2 reference pattern JCPDS/PDF #99-0088.

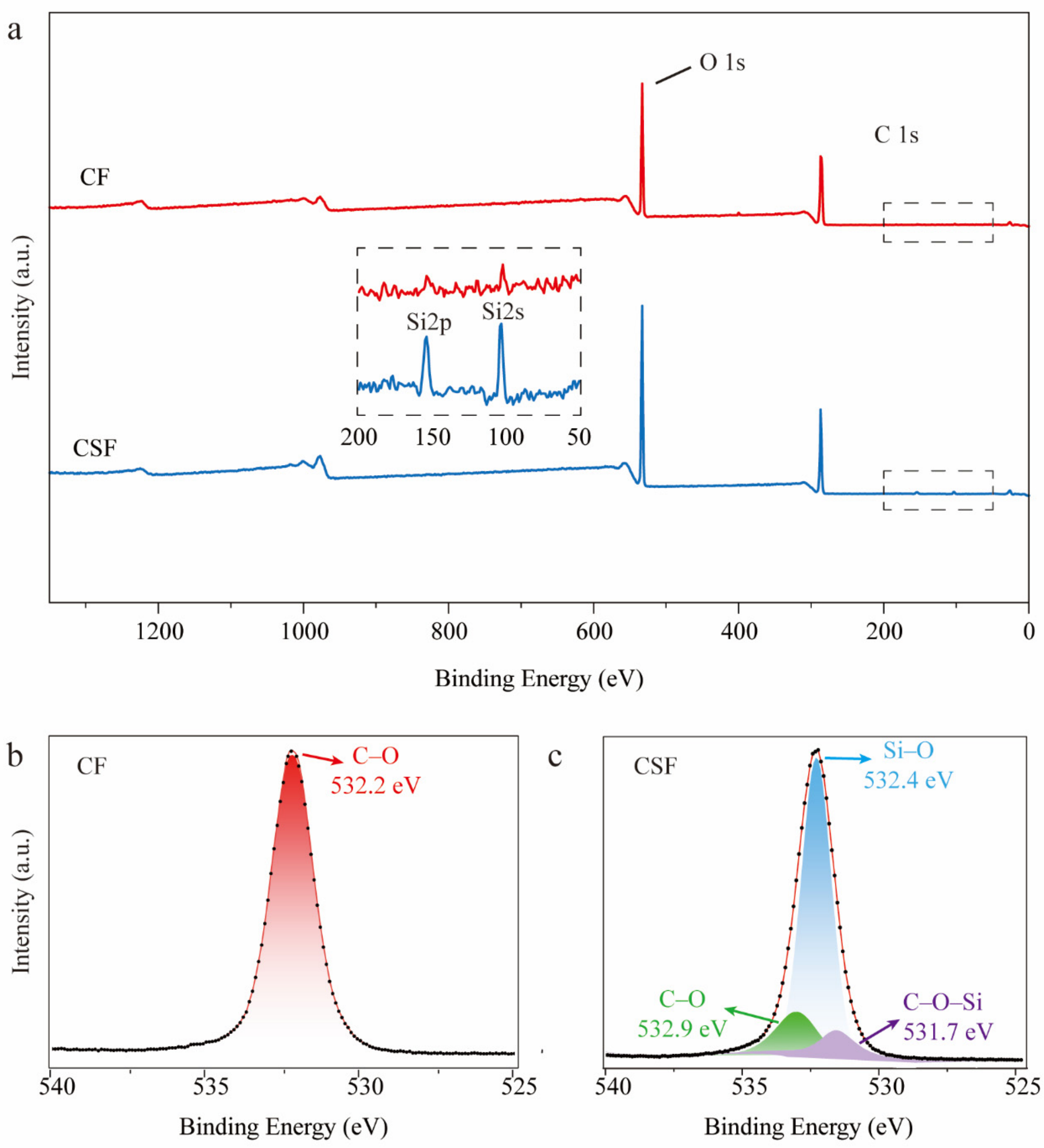

Figure S6. XPS characterization of cellulose fiber (CF) and CSF. (a) Survey XPS spectra of CF and CSF. Both samples show characteristic C 1s and O 1s signals, while CSF additionally exhibits Si 2p and Si 2s signals in the enlarged inset, confirming the successful incorporation of SiO_2 into the fiber system. (b) High-resolution O 1s spectrum of CF, showing a dominant C–O-related oxygen component centered at 532.2 eV. (c) High-resolution O 1s spectrum of CSF, which can be deconvoluted into Si–O-related oxygen at 532.4 eV, C–O-related oxygen at 532.9 eV, and an interfacial C–O–Si-related oxygen component at 531.7 eV.

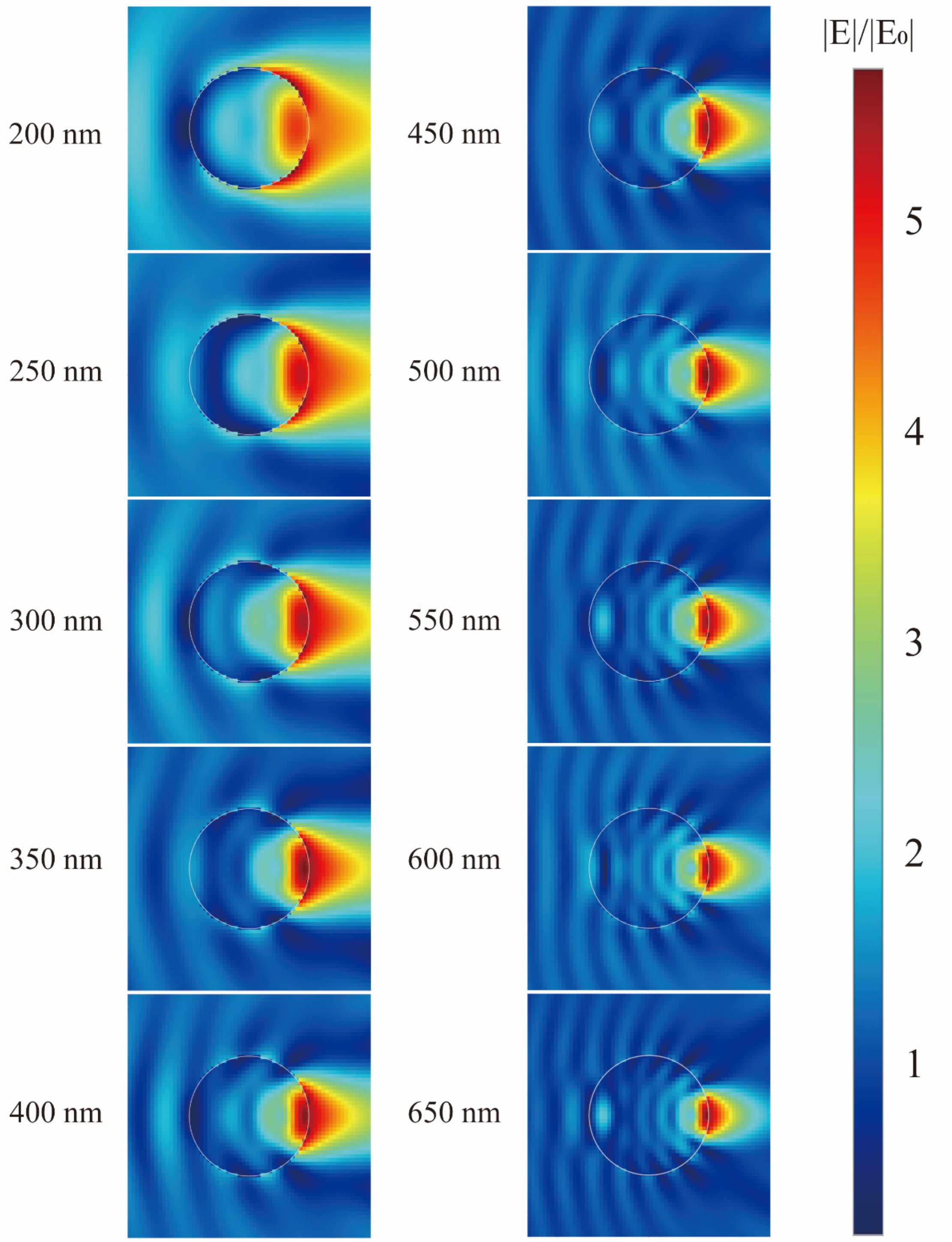

Figure S7. FDTD simulated electric field distributions ($|E|/|E_0|$) of nano-SiO_2 (200–650 nm). The optimal Mie scattering resonance is observed at a diameter of 500 nm, justifying its use to maximize solar reflectance.

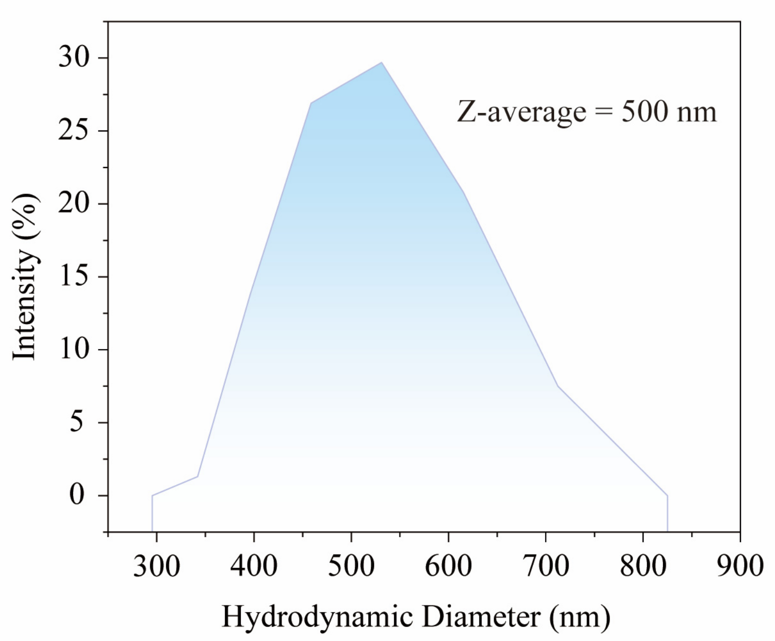

Figure S8. Intensity-weighted hydrodynamic particle-size distribution of the commercial SiO_2 dispersion measured by dynamic light scattering.

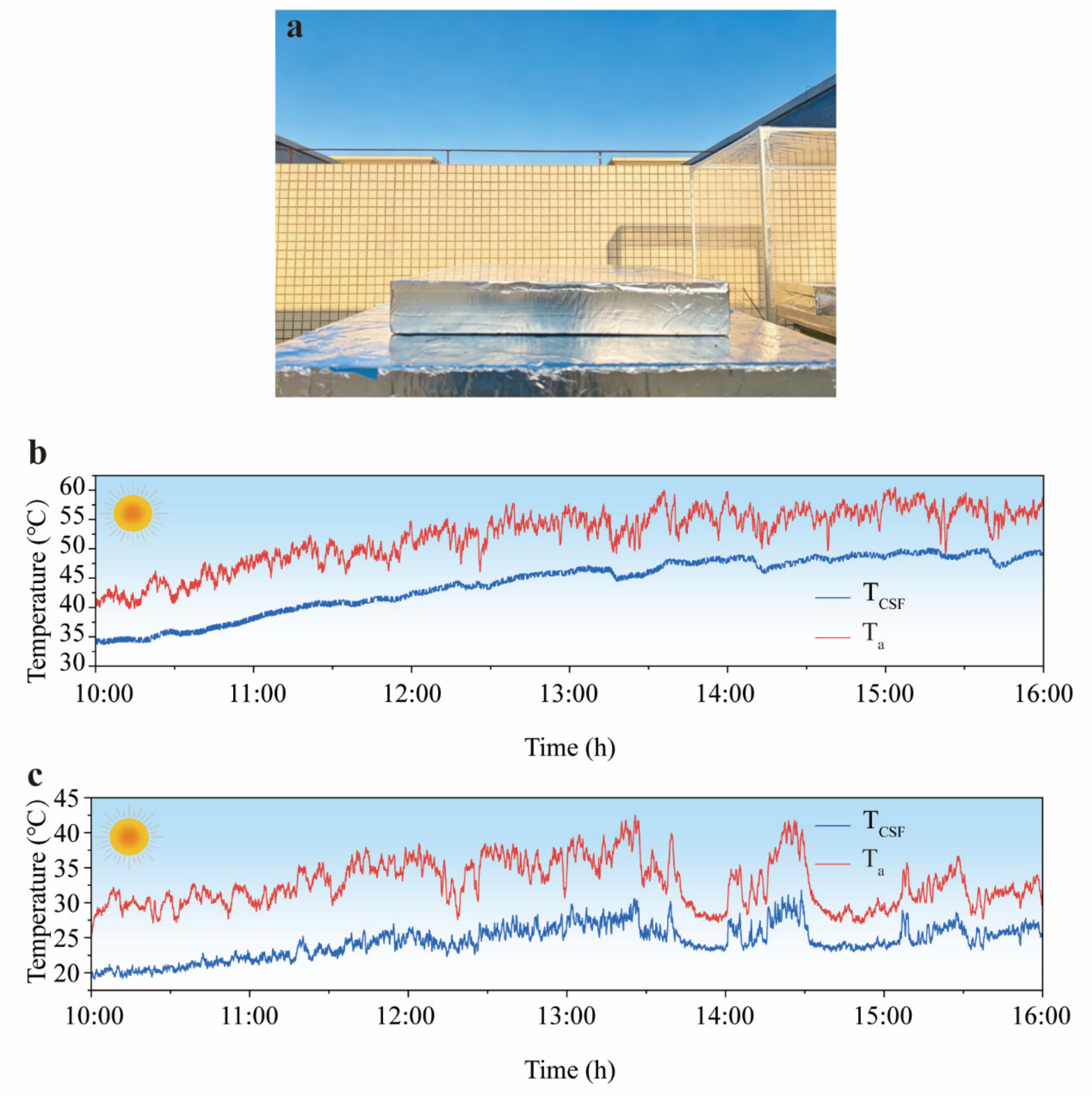

Figure S9. Supplementary continuous outdoor cooling performance of CSF. (a) Photograph of the outdoor experimental setup used for evaluating the cooling performance. The sample is placed on a thermally insulated platform under natural sunlight. (b, c) Daytime temperature profiles of CSF in Fuzhou, China, on (b) 15 October 2025 and (c) 15 November 2025.

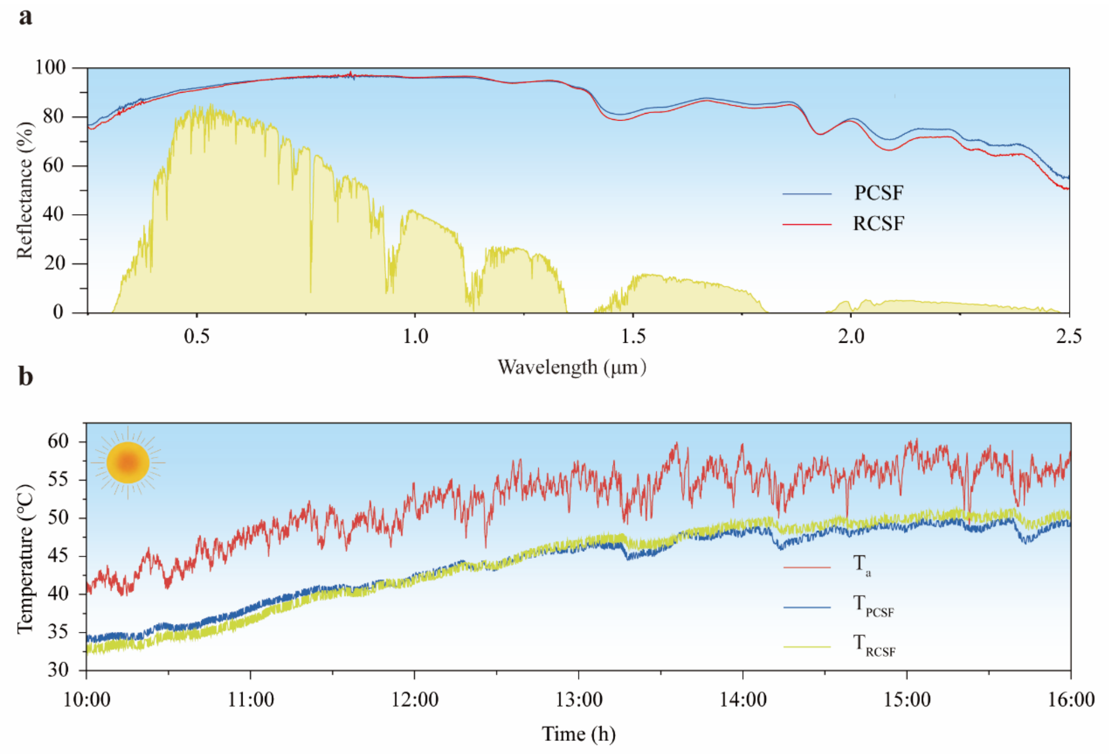

Figure S10. Light and outdoor cooling performance retention of recycled CSF. (a) Reflectance spectra of pristine CSF (PCSF) and recycled CSF (RCSF). PCSF exhibits an average reflectance of 94.56% in the 0.4–1.0 μm region, while RCSF retains an average reflectance of 94.45% in the same region, corresponding to 99.88% retention relative to PCSF. (b) Outdoor temperature profiles of ambient air (T_a), PCSF (T_{PCSF}), and RCSF (T_{RCSF}) in Fuzhou, China, on 15 October 2025, from 10:00 to 16:00. RCSF maintains an average daytime cooling of 8.02 °C, close to that of PCSF (8.23 °C), indicating that CSF largely preserves its light-reflectance and radiative-cooling capabilities after closed-loop reuse.
